# The Ten-Item Personality Inventory (TIPI): a scoping review of versions, translations and psychometric properties

**DOI:** 10.3389/fpsyg.2023.1202953

**Published:** 2023-06-26

**Authors:** Mikkel M. Thørrisen, Talieh Sadeghi

**Affiliations:** ^1^Department of Rehabilitation Science and Health Technology, Faculty of Health Sciences, OsloMet – Oslo Metropolitan University, Oslo, Norway; ^2^Department of Public Health, Faculty of Health Sciences, University of Stavanger, Stavanger, Norway; ^3^Work Research Institute, Centre for Welfare and Labour Research, OsloMet – Oslo Metropolitan University, Oslo, Norway

**Keywords:** Big Five, convergent validity, internal consistency, personality assessment, psychometric properties, structural validity, ten-item personality inventory, test–retest reliability

## Abstract

**Background:**

The Ten-Item Personality Inventory (TIPI) is a brief instrument designed to assess the five-factor model (FFM) personality dimensions. It was specifically developed to provide a brief assessment option in situations where using more comprehensive FFM instruments would be unfeasible. The TIPI enjoys widespread use and has been translated into several different languages.

**Objective:**

The aim of this scoping review was to generate an overview of different versions of the TIPI, and their psychometric properties in terms of two aspects of validity (convergent and structural) and two aspects of reliability (internal consistency and test–retest reliability).

**Methods:**

Four databases (PsycINFO, PubPsych, Medline, and Web of Science) were searched for studies exploring psychometric properties of the TIPI (original and/or translated or revised versions), published in English as full-text original research articles. Additionally, manual searches were conducted on the official TIPI website and in reference lists. Studies who utilized the TIPI simply as a measure, without an aim of testing its psychometric properties, were excluded. A descriptive-analytical approach was utilized to generate overviews of available TIPI versions and their psychometric properties.

**Results:**

In a total of 29 studies, 27 versions of the TIPI were identified, covering 18 different languages. Across versions, and evaluated against conventions of acceptable psychometric properties, the TIPI demonstrated acceptable test–retest reliability, somewhat mixed results for convergent and structural validity, and inappropriate internal consistency.

**Conclusion:**

Being a brief instrument, the TIPI is unsurprisingly characterized by certain psychometric shortcomings. However, the TIPI may represent a feasible compromise in instances where it is necessary to strike a balance between maximizing psychometric properties and minimizing survey length.

## 1. Introduction

Personality refers to “psychological qualities that contribute to an individual’s enduring and distinctive pattern of feeling, thinking, and behaving” (Cervone and Pervin, 2013, p. 8), and thus to “individual differences, or the dimensions along which people differ from each other” (Barenbaum and Winter, 2008, p. 7). The first psychological literature review of personality emerged in 1921 (Allport, 1921), and personality has since been a fundamental concept in psychology. Research has demonstrated that personality traits predict a variety of outcomes, such as health, longevity (Ozer and Benet-Martinez, 2006; Roberts et al., 2007), job performance and satisfaction (Judge and Bono, 2001), consumption of psychoactive substances (Paunonen and Ashton, 2001), political participation (Mondak and Halperin, 2008), economic and social policy attitudes (Gerber et al., 2010), wage and occupational self-efficacy (Sadeghi et al., 2022), and traffic behavior (Ulleberg and Rundmo, 2003). Personality is characterized by a cross-disciplinary applicability, by not solely being of interest within psychology, but in all research areas that on some level deal with human factors (Roberts et al., 2007), such as sociology, education and business. In research studies, personality traits may be utilized as predictors or outcomes (the study variables of primary interest), as mediators or moderators, or simply as important control variables when studying relationships between other constructs.

The five-factor model (FFM) of personality, also known as the Big Five personality model, is the leading framework for understanding individual differences (McCrae and Costa, 2008; Sprecht et al., 2014). According to the FFM, personality is composed of five broad domains: extraversion (E, being sociable and active), agreeableness (A, being soft-hearted and trusting), conscientiousness (C, being organized and reliable), emotional stability (ES^1^, being calm and relaxed), and openness (O, being curious and creative) (Costa and McCrae, 1992). The FFM has been shown to be quite consistent across cultures (McCrae et al., 1998).

Several instruments have been developed to measure the five domains of the FFM, of which the 240-item NEO Personality Inventory-Revised (NEO-PI-R, Costa and McCrae, 1992), the 60-item NEO Five-Factor Inventory (NEO-FFI, Costa and McCrae, 1992), and the 44-item Big Five Inventory (BFI, John and Srivastava, 1999) are the most prominent. Due to their number of items, these FFM instruments are impractical to administer in studies that do not aim to answer research questions primarily related to personality (DeBell et al., 2022). The most extensive tool (the NEO-PI-R) takes about 45 min to complete (Gosling et al., 2003). Even the NEO-FFI and the BFI, which take 15 and 5 min to complete, respectively (John and Srivastava, 1999), may be too comprehensive in many instances. Long surveys may reduce participation rates (Rolstad et al., 2011), due to respondents being bored or fatigued (Credé et al., 2012), which may result in data quality decrements. This represents a particular concern for longitudinal studies that require repeated measurements (Herzberg and Brähler, 2006).

The need for brief FFM instruments is evident, particularly in instances when researchers want to study personality as just one out of several other constructs, or utilize personality traits as control variables. In 2003, Samuel Gosling and coworkers introduced the Ten-Item Personality Inventory (TIPI) as a brief instrument to assess the FFM personality dimensions (Gosling et al., 2003). The TIPI consists of 10 items, with five two-item subscales that correspond to the FFM dimensions. The initial evaluation of the TIPI (Gosling et al., 2003) found that it was an appropriate alternative to more extensive FFM instruments, with acceptable validity, reliability, and external correlations. For several reasons, the TIPI stands out as appealing to researchers: It is freely available (Storme et al., 2016), and it takes approximately just 1 min to complete (Nunes et al., 2018), thereby extending the scope of studies in which FFM personality dimensions can be taken into account (Gosling et al., 2003). The evident appeal of the TIPI is reflected in its widespread use, as the initial validation article (Gosling et al., 2003) has been cited more than 9,500 times in the research literature. Moreover, according to the official TIPI website (Gosling, n.d.), the original instrument has been translated into at least 26 different languages for worldwide utilization.

However, as noted by Ehrhart et al. (2009), the interest in utilizing the TIPI quickly outpaced the efforts spent on investigating the instrument’s psychometric properties. Psychometric properties refer to the degree of validity and reliability associated with a measurement instrument (Asunta et al., 2019). Validity represents the degree to which an instrument measures the construct(s) it purports to measure (Mokkink et al., 2010, p. 743). Structural and convergent validity, both elements of construct validity, are of particular importance. Scores on the instrument should represent an adequate reflection of the dimensionality of the construct to be measured (structural validity), and scores on the instrument should correlate with scores on other instruments designed to measure the same construct (convergent validity) (Cunningham et al., 2001; Mokkink et al., 2010). The concept of reliability captures the extent to which an instrument is free from measurement error, which is often expressed in terms of the degree of interrelatedness of the items (internal consistency) and the instrument’s ability to generate similar results when repeatedly administered to the same respondents under the same conditions (test–retest reliability) (Mokkink et al., 2010; Noble et al., 2021).

Testing of psychometric properties such as internal consistency and structural validity rests on an assumption of the measure being reflective rather than formative. Reflective measures presume that the test items are caused by a common latent variable, while formative measures postulate that the construct being measured represents a function of the items, rather than the other way around (Markus and Borsboom, 2013). For instance, in the case of the E subscale in the TIPI, one could argue that being sociable and active are behaviors caused by high levels of extraversion (reflective approach), or alternatively that high extraversion is a function of being sociable and active (formative approach). Being developed on the basis of reflective techniques (such as factor analysis), the FFM is generally measured by means of reflective approaches (McCrae and John, 1992).

Comprehensive instruments tend to have better psychometric properties than shorter instruments (Gosling et al., 2003). For instance, short scales generally suffer from not being able to suppress random error through aggregation of items (Herzberg and Brähler, 2006), and from being characterized by content deficiencies (Smith et al., 2000). However, some studies indicate that psychometric shortcomings associated with brief instruments may not be severe enough to discourage the use of short scales in research altogether (Credé et al., 2012). In practice, researchers have to make a trade-off and strike an appropriate balance between maximizing psychometric properties on the one hand, and minimizing administration time and survey length on the other (Furnham, 2008). As noted by Gosling et al. (2003, p. 505), “researchers may be faced with a stark choice of using an extremely brief instrument or using no instrument at all.”

Authors have emphasized the need for further attention to psychometric properties of the TIPI (Ehrhart et al., 2009), and for secondary research efforts to provide an overview of different versions of the TIPI and their psychometric properties (Thørrisen et al., 2021). The aim of the current scoping review was to generate an overview of different versions of the TIPI, and their psychometric properties in terms of two aspects of validity (convergent and structural) and two aspects of reliability (internal consistency and test–retest reliability).

## 2. Materials and methods

### 2.1. Design and protocol

The study was designed as a scoping review, following the methodology first established by Arksey and O'Malley (2005), and further developed by others (Levac et al., 2010; Colquhoun et al., 2014; Peters et al., 2020). The review was pre-registered in the Open Science Framework (OSF) database (Thørrisen and Sadeghi, 2023), and reported in accordance with the PRISMA extension for scoping reviews (PRISMA-ScR) (Tricco et al., 2018).

### 2.2. Eligibility criteria

For inclusion in this scoping review, studies had to satisfy three criteria. First, studies had to explore psychometric properties of the TIPI (original and/or translated or revised version) as a study aim. Selection of relevant psychometric properties for this review was inspired by the COSMIN taxonomy of relationships of measurement properties (Mokkink et al., 2010), and included two aspects of validity (structural and convergent) and two aspects of reliability (internal consistency and test–retest reliability). A test of convergent validity was defined as a correlation between the TIPI subscales (original, revised or translated version) with subscales from NEO-PI-R, NEO-FFI and/or BFI. These instruments were chosen as a result of being the most prominent and most commonly used to measure the FFM dimensions. Studies who utilized the TIPI simply as a measure (predictor, outcome or covariate) without an aim of exploring psychometric properties were excluded. Second, studies had to be published as full-text original peer-reviewed research articles. Third, studies had to be published in English. No time restrictions were imposed.

### 2.3. Literature search

The superordinate search strategy for this scoping review comprised three parts: (i) systematic searches in four international scientific databases, (ii) manual searches on the TIPI website, and (iii) manual searches in reference lists of all included studies. Searches were initially conducted in February 2023.

#### 2.3.1. Database searches

The database search strategy focused on two search blocks, one denoting the instrument itself, and one capturing relevant psychometric properties. The primary database search strategy is presented in Table 1.

**Table 1:** Database search strategy (PsycINFO).

Concept		Search terms
Concept 1	The instrument (the Ten-Item Personality Inventory)	“ten-item personality inventory.”tw OR “ten item personality inventory.”tw OR tipi.tw OR “10-item personality inventory.”tw OR “10 item personality invenfory.”tw OR “10-item measure of the big-five dimensions.”tw
		AND
Concept 2	Psychometric properties	Psychometrics[sh] OR measurement[sh] OR cross cultural validity[sh] OR factorial validity[sh] OR content validity[sh] OR discriminant validity[sh] OR construct validity[sh] OR test validity[sh] OR convergent validity[sh] OR face validity[sh] OR statistical validity[sh] OR criterion validity[sh] OR predictive validity[sh] OR split-half reliability[sh] OR test–retest reliability[sh] OR interrater reliability[sh] OR test reliability[sh] OR statistical reliability[sh] OR internal consistency[sh] OR factor analysis[sh] OR principal component analysis[sh] OR psychometric*.tw OR measurement*.tw OR develop*.tw OR valid*.tw OR reliab*.tw OR “internal consistency.”tw OR “test–retest.”tw OR “test retest.”tw OR “factor analys*.”tw OR factorial.tw OR “principal component*.”tw OR pca.tw OR adapt*.tw OR translat*.tw

.tw, text search on title/abstract level; [sh], search based on subject heading (APA thesaurus of psychological index terms); searches were also conducted in three other databases (PubPsych, Medline, Web of Science), with necessary adjustments for each database.

As shown in Table 1, the database search strategy comprised a total of 45 searches, of which 21 were text searches on an abstract/title level, 21 were searches on subject headings (APA thesaurus of psychological index terms), and the remaining 3 were combinations of search blocks utilizing Boolean operators (OR; AND). Searches were performed in four scientific databases. PsycINFO and PubPsych were defined as the primary databases. In order to reflect the cross-disciplinary applicability of personality research, searches were also conducted in databases emphasizing health science research (Medline) and social science research (Web of Science). Where necessary, the search strategy was adjusted to fit each database.

#### 2.3.2. Manual searches

The TIPI website (Gosling, n.d.) is developed and maintained by Samuel Gosling, the developer of the TIPI. Although not completely updated, the website contains general information about the instrument, an overview of translated versions, and links to studies that have explored psychometric properties of different versions. The TIPI website was searched manually for potentially relevant studies not identified in database searches. Additionally, reference lists in included studies were searched manually for potentially relevant studies not identified in database searches or searches on the TIPI website.

### 2.4. Study selection

First, results from database searches were exported from databases to EndNote version 20 (Clarivate Analytics, 2020). Duplicates were removed and the unique records (titles and abstracts) were transferred to Rayyan, a web-based software platform for literature reviews (Qatar Computing Research Institute, 2021). Unique records were screened for relevance in Rayyan on a title/abstract level. Studies deemed potentially relevant (based on the title/abstract screening) were retrieved in full-text format for further inspection. Primary reason for exclusion was registered for studies excluded in the full-text examination stage. The full-text examination was documented in Excel 365 (Microsoft Corporation, 2021). Study selection based on database search results was performed independently by the two authors. Initial disagreements were resolved through discussion, and consensus was reached.

Second, studies listed on the TIPI website were inspected manually. Potentially relevant studies, that were not already identified through database searches, were assessed for eligibility. A two-step process was utilized: screening on a title/abstract level, followed by a full-text examination. Third, reference lists in all included studies (from databases and the website) were screened for relevance based on titles. Potentially relevant titles, not already identified through databases and the website, were screened on an abstract level. Titles not excluded in the screening stage, were retrieved and inspected in full-text format.

### 2.5. Data charting and data items

Two types of information were extracted from the included studies: (i) data on study and inventory characteristics (title, author(s), year of publication, sample characteristics and size, TIPI version) and (ii) data and results on psychometric properties (convergent validity, structural validity, internal consistency, and test–retest reliability). Extracted data were entered into a data extraction form for further analysis, generated in Excel 365 (Microsoft Corporation, 2021). Data extraction was conducted by the first author, and a random selection of 8 studies (approximately 25% of the studies) was cross-checked by the second author.

### 2.6. Synthesis of results

Data analysis was conducted in accordance with a descriptive-analytical procedure recommended for scoping reviews (Arksey and O'Malley, 2005; Levac et al., 2010). First, a descriptive overview of different versions of the TIPI (and studies that have explored their psychometric properties) was constructed. Each version was ascribed an ID consisting of a three-letter code based on the ISO 639-2 language code system (Library of Congress, 2010). In instances of several versions in the same language, each version was also provided with a number (e.g., ENG-1 and ENG-2, indicating two different versions in English).

Second, an overview of which psychometric properties have been tested for each version of the TIPI was generated. In order to illustrate the extent to which each version has been subjected to psychometric inspection, a simple scoring system was developed. For each study testing a particular version, the TIPI version was given a score 1–4, corresponding to the number of psychometric domains tested. For instance, a version was given a score of 2 if it was tested on two domains in one study (e.g., internal consistency and structural validity). If a version was tested on two domains in one study, on three domains in another, and on one domain in a third study, the overall ascribed score for the version would be 2 + 3 + 1 = 6.

Third, results of psychometric tests reported in the included studies were synthesized. *Convergent validity* of the TIPI versions was presented by means of reported convergence (correlations) with subscales in three other validated FFM instruments (NEO-PI-R, NEO-FFI, and BFI). Additionally, mean estimates for convergence across all five dimensions were calculated, separately for each TIPI version and for all versions taken together. Acceptable convergent validity was *a priori* defined as *r* ≥ 0.50 (Cohen, 1988; Abma et al., 2016). Regarding *structural validity*, each TIPI version was classified based on the extent to which it has demonstrated a five-factor structure theoretically in accordance with the FFM (“yes,” “no” or “partly”). A “partly” satisfactory structure was defined as identifying an acceptable five-factor structure after making certain statistical adjustments. *Internal consistency* of the TIPI versions was reported in terms of Cronbach’s alphas (α), inter-item correlations (*r*) and/or Spearman-Brown coefficients (S-B). Mean estimates for each version, for each subscale across versions, and for all five subscales across versions were calculated based on subscale values reported in the included studies. Acceptable internal consistency was defined as α ≥ 0.70 (Nunnally, 1978), *r* ≥ 0.50 (Cohen, 1988) or S-B ≥ 0.70 (Salkind, 2010). *Test–retest reliability* of the TIPI versions was presented in terms of test–retest correlations reported for each subscale in the included studies. Mean estimates for temporal stability across all five dimensions were calculated, separately for each TIPI version, and for all versions taken together. Acceptable test–retest reliability was defined as *r* ≥ 0.50 (Cohen, 1988) or intraclass correlation (ICC) ≥0.60 (Cicchetti, 1994).

Finally, a summative evaluation of psychometric properties associated with each of the identified TIPI versions was generated.

## 3. Results

**Figure 1:**
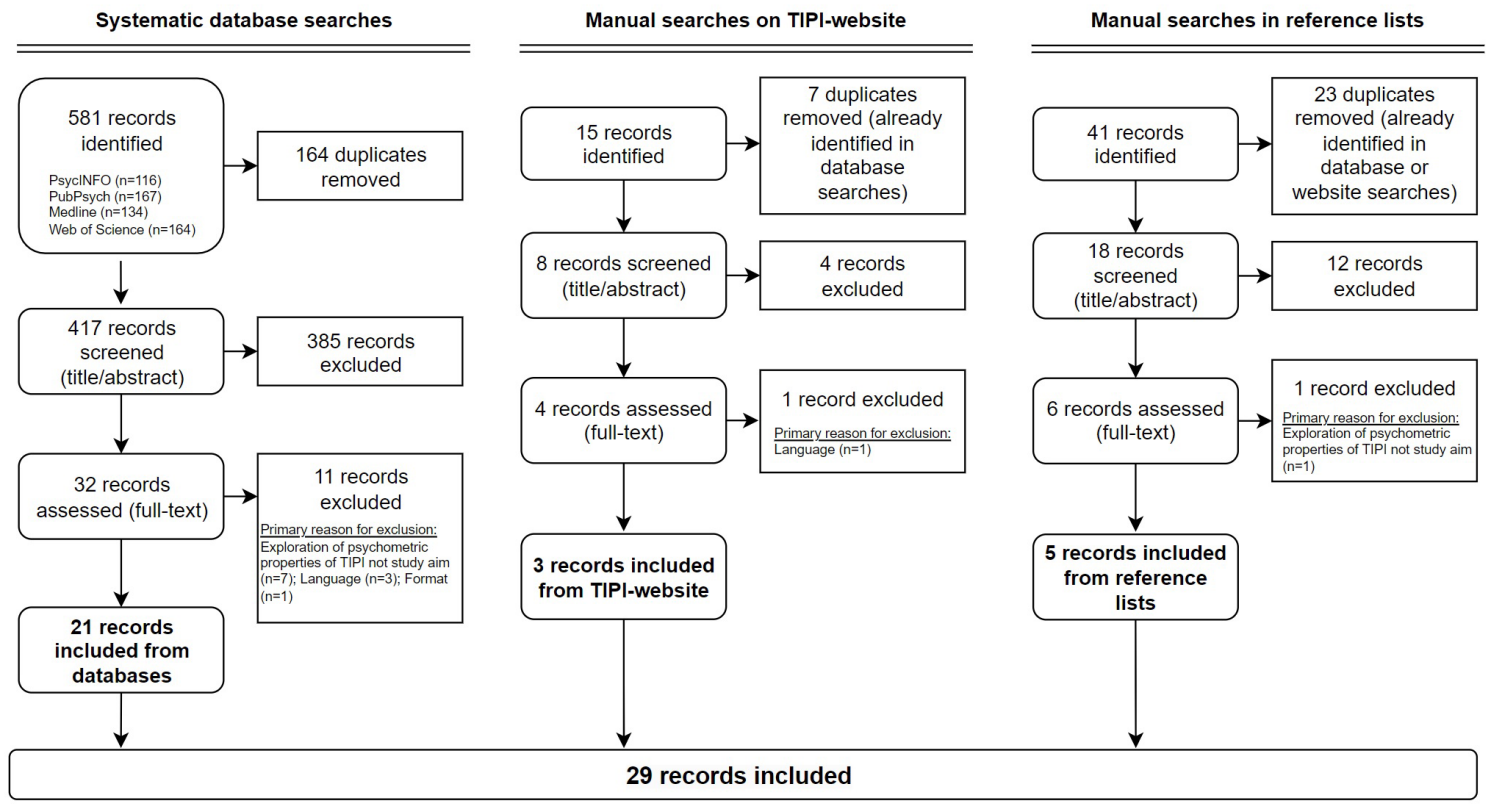
Flow chart depicting the study selection process.

Database searches identified 581 studies (PsycINFO: *n* = 116; PubPsych: *n* = 167; Medline: *n* = 134; Web of Science: *n* = 164). Of these, 164 duplicates were removed and 417 unique studies were screened on a title/abstract level. A total of 385 studies were excluded due to not fulfilling the eligibility criteria, and the remaining 32 studies were subjected to full-text examination. At this stage, another 11 studies were excluded as a result of not having exploration of psychometric properties of the TIPI as a study aim (*n* = 7), due to not being published in English (*n* = 3), or not being published as a peer-reviewed research article (*n* = 1). Hence, 21 studies from database searches were included in the scoping review. Manual searches on the TIPI website and in reference lists resulted in an additional eight studies being included (website: *n* = 3; reference lists: *n* = 5). In total, 29 studies were included in the scoping review. The study selection process is illustrated in Figure 1.

The 29 included studies were based on a total of 27,427 participants from 20 countries (Bangladesh, Brazil, China, Croatia, France, Georgia, Germany, Indonesia, Iran, Italy, Japan, Netherlands, Norway, Poland, Portugal, South Africa, Spain, Turkey, the United Kingdom, and the United States), published from 2003 (Gosling et al., 2003) to 2022 (DeBell et al., 2022; Shi et al., 2022). Sample sizes ranged from *N* = 100 (Furnham, 2008) to *N* = 5,009 (Thørrisen et al., 2021). Characteristics of the included studies are presented in Table 2.

**Table 2:** Characteristics of the included studies (*N* = 29).

Author(s) (year)	Country	Sample size	Sample characteristics
Akhtar (2018)	Indonesia	*N* = 501	High school/university students; 68.1% females; age 15–40 years (*M* = 19.2)
Atak (2013)	Turkey	*N* = 420	University students (age *M* = 23.2 years) and non-students (age *M* = 23.4 years)
Azkhosh et al. (2020)	Iran	*N* = 160	Older individuals (>60 years); 57% males
Brito-Costa et al. (2015)	Portugal	*N* = 170	Male soccer athletes; age 13–33 years (*M* = 18.5)
Carvalho et al. (2012)	Brazil	*N* = 404	Secondary school students; 59.4% females; age 14–20 years (*M* = 15.9)
Chiorri et al. (2015)	Italy	*N* = 884	Study 1 (*n* = 189): general population (72% females, age 18–65 years); Study 2 (*n* = 157): university students (78% females, age 18–42 years); Study 3 (*n* = 472): General population (52% female, age 18–77 years); Study 4 (*n* = 66): university students (82% females, age 19–59 years)
Credé et al. (2012)	USA	*N* = 832	Sample 1 (*n* = 437): employees (51% females, age *M* = 40.1); Sample 2 (*n* = 395): university students (58% females, age *M* = 19.1)
DeBell et al. (2022)	USA	*N* = 2,816	Two general population probabily samples (sample 1: *n* = 1,253, sample 2: *n* = 1,563)
Denissen et al. (2008)	Netherlands	*N* = 221	University students; 82% females; age *M* = 18.9 years
Ehrhart et al. (2009)	USA	*N* = 902	University students; 51% females; age *M* = 21.8 years
Furnham (2008)	United Kingdom	*N* = 100	University students; 78% females; age *M* = 19.7 years
Gosling et al. (2003)	USA	*N* = 1,813^a^	University students; 65% females
Herzberg and Brähler (2006)	Germany	*N* = 2,916^a^	Sample 1 (*n* = 2,552): general population [53% females, age 14–99 years (*M =* 47.6)]; Sample 2 (*n* = 364): friends/relatives of participants in sample 1 [54.9% females, age 18–94 years (*M* = 13.8)]
Hofmans et al. (2008)	Netherlands	*N* = 345	University students; 77.5% females; age 13–63 years (*M* = 18.5)
Islam (2019)	Bangladesh	*N* = 662	General population; 53.8% females; age 15–60 years (*M* = 43.0)
Iwasa and Yoshida (2018)	Japan	*N* = 832	Sample 1 (*n* = 520): middle-aged adults (40–64 years), 50% females; Sample 2 (*n* = 312): older adults (65–79 years), 50% females
Laguna et al. (2014)	Poland	*N* = 399	University students; 64.4% females; age 18–34 years (*M* = 22.0)
Martskvishvili et al. (2020)	Georgia	*N* = 377^a^	Students; 44.6% females; age 16–58 (*M* = 20.6)
Metzer et al. (2014)	South Africa	*N* = 662	University students; 80% females; age *M* = 21.0 years
Muck et al. (2007)	Germany	*N* = 180	General population; 48.3% females; age 17–75 years (*M* = 25.0)
Nunes et al. (2018)	Portugal	*N* = 333	University students; 78% females; age *M* = 33.2
Oshio et al. (2013)	Japan	*N* = 163	University students; 58.9% females; age *M* = 19.0
Oshio et al. (2014)	Japan	*N* = 228	University students; 62.7% females; age *M* = 19.5
Renau et al. (2013)	Spain	*N* = 500	University students and volunteers recruited by students (study 1: *n* = 309; study 2: *n* = 191)
Romero et al. (2012)	Spain	*N* = 1,181	University students and adults from the general population; 62.3% females; age 21–61 years (*M* = 35.6)
Schult et al. (2019)	Germany	*N* = 198	University students; 75% females; age 18–45 years (*M* = 23.2)
Shi et al. (2022)	China	*N* = 2,223	University students; 74.1% females; age *M* = 22.5 years
Storme et al. (2016)	France	*N* = 1,554	Students and adults from the general population; 59.3% females; age 16–88 years (*M* = 28.6)
Thørrisen et al. (2021)	Norway	*N* = 5,009	Graduates with a master degree; 62.4% females; the majority aged 28–30 years
Vorkapic (2016)	Croatia	*N* = 432	Teachers; 99.1% females; age 22–64 (*M* = 39.1)

a Subsample involved in psychometric testing of the Ten-Item Personality Inventory (TIPI) in studies consisting of a larger total sample; *M*, mean.

### 3.1. TIPI versions and studies exploring their psychometric properties

A total of 27 versions of the TIPI were identified in the 29 included studies. These versions covered 18 different languages, i.e., English (Gosling et al., 2003; Furnham, 2008; Ehrhart et al., 2009; Credé et al., 2012; Metzer et al., 2014; DeBell et al., 2022), Bengali (Islam, 2019), Catalan (Renau et al., 2013), Chinese (Shi et al., 2022), Croatian (Vorkapic, 2016), Dutch (Denissen et al., 2008; Hofmans et al., 2008), French (Storme et al., 2016), German (Herzberg and Brähler, 2006; Muck et al., 2007; Schult et al., 2019), Georgian (Martskvishvili et al., 2020), Indonesian (Akhtar, 2018), Italian (Chiorri et al., 2015), Japanese (Oshio et al., 2013, 2014; Iwasa and Yoshida, 2018), Norwegian (Thørrisen et al., 2021), Persian (Azkhosh et al., 2020), Polish (Laguna et al., 2014), Portuguese (Carvalho et al., 2012; Brito-Costa et al., 2015; Nunes et al., 2018), Spanish (Romero et al., 2012; Renau et al., 2013), and Turkish (Atak, 2013). An overview and description of the TIPI versions and studies exploring their psychometric properties are presented in Table 3.

**Table 3:** Overview and descriptions of versions of the TIPI and studies exploring their psychometric properties.

Version^a^	Language	Abbreviation^b^	Description	Studies^c^
ENG-1	English	TIPI	Original TIPI	Gosling et al. (2003)
				Furnham (2008)
				Ehrhart et al. (2009)
				Credé et al. (2012)
				Metzer et al. (2014)
				DeBell et al. (2022)
ENG-2	English		Revised version of ENG-1. Uses shorter instruction and uses verbal labels for response options instead of numbers	DeBell et al. (2022)
BEN	Bangla/Bengali	TIPI-B	Bangla/Bengali translation of ENG-1	Islam (2019)
CAT	Catalan	TIPI-CAT	Catalan translation of ENG-1	Renau et al. (2013)
CHI	Chinese		Chinese translation of ENG-1	Shi et al. (2022)
DUT-1	Dutch	TIPI-d v1	Dutch translation of ENG-1	Hofmans et al. (2008)
DUT-2	Dutch	TIPI-d v2	Revised version of DUT-1. Revised wording of five items	Hofmans et al. (2008)
DUT-3	Dutch	TIPI-r	Dutch translation of ENG-1, with bipolar rating scales	Denissen et al. (2008)
FRE	French		French translation of ENG-1	Storme et al. (2016)
GER-1	German		German translation of ENG-1	Herzberg and Brähler (2006)
GER-2	German	TIPI-G	German translation of ENG-1	Muck et al. (2007)
				Schult et al. (2019)
GER-3	German		Revised version of GER-2. Single-descriptor instead of multi-descriptor items (20 instead of 10 items)	Schult et al. (2019)
GEO	Georgian		Georgian translation of ENG-1	Martskvishvili et al. (2020)
HRV	Croatian		Croatian translation of ENG-1	Vorkapic (2016)
IND	Indonesian		Indonesian translation of ENG-1	Akhtar (2018)
ITA-1	Italian	I-TIPI	Italian translation of ENG-1	Chiorri et al. (2015)
ITA-2	Italian	I-TIPI-R	Revision of ITA-1. Wording of some items revised	Chiorri et al. (2015)
JPN	Japanese	TIPI-J	Japanese translation of ENG-1	Oshio et al. (2013, 2014)
				Iwasa and Yoshida (2018)
NOR	Norwegian	N-TIPI	Norwegian translation of ENG-1	Thørrisen et al. (2021)
PER	Persian		Persian translation of ENG-1	Azkhosh et al. (2020)
POL	Polish	TIPI-P	Polish translation of ENG-1	Laguna et al. (2014)
POR-1	Portuguese (Brazil)	TIPI-Br	Portuguese (Brazil) translation of ENG-1	Carvalho et al. (2012)
POR-2	Portuguese		Portuguese translation of ENG-1	Brito-Costa et al. (2015)
				Nunes et al. (2018)
SPA-1	Spanish		Spanish translation of ENG-1	Romero et al. (2012)
SPA-2	Spanish	TIPI-SPA	Spanish translation of ENG-1. Based on SPA-1	Renau et al. (2013)
SPA-3	Spanish	TIPI-SPA-v2	Revised version of SPA-2. Revised wording of two items	Renau et al. (2013)
TUR	Turkish		Turkish translation of ENG-1	Atak (2013)

a Code consisting of ISO 639-2 three-letter language code and number indicating language version.

b Instrument abbreviation utilized in studies exploring psychometric properties of the instrument.

c Studies that have explored psychometric properties of the version.

### 3.2. Psychometric properties tested for the TIPI versions

Based on the 29 included studies, the original TIPI (ENG-1) was subjected to the most extensive psychometric inspection, with all four psychometric domains (convergent validity, structural validity, internal consistency and test–retest reliability) tested in six studies (Gosling et al., 2003; Furnham, 2008; Ehrhart et al., 2009; Credé et al., 2012; Metzer et al., 2014; DeBell et al., 2022). The Japanese translation of the TIPI (JPN) was tested on four domains in three studies (Oshio et al., 2013, 2014; Iwasa and Yoshida, 2018), the second Portuguese version (POR-2) was tested on four domains in two studies (Brito-Costa et al., 2015; Nunes et al., 2018), and the second German version (GER-2) was tested on three domains in two studies (Muck et al., 2007; Schult et al., 2019). Across all versions of the TIPI, internal consistency was most frequently tested (*n* = 28 tests), followed by convergent validity (*n* = 26 tests), structural validity (*n* = 22 tests) and test–retest reliability (*n* = 17 tests). An overview of psychometric properties tested for the TIPI versions is presented in Table 4.

**Table 4:** Overview of psychometric properties tested for the TIPI versions.

		Validity		Reliability		
Version^a^	Study	Con.^b^	Str.	IC	T-R	Score^c^
ENG-1	Gosling et al. (2003)	●		●	●	11
	Furnham (2008)	●				
	Ehrhart et al. (2009)		●	●		
	Credé et al. (2012)			●		
	Metzer et al. (2014)		●	●		
	DeBell et al. (2022)			●		
ENG-2	DeBell et al. (2022)			●	●	2
BEN	Islam (2019)	●	●	●	●	4
CAT	Renau et al. (2013)	●		●	●	3
CHI	Shi et al. (2022)		●	●		2
DUT-1	Hofmans et al. (2008)	●	●			2
DUT-2	Hofmans et al. (2008)	●	●			2
DUT-3	Denissen et al. (2008)	●			●	2
FRE	Storme et al. (2016)	●	●	●	●	4
GER-1	Herzberg and Brähler (2006)	●	●	●	●	4
GER-2	Muck et al. (2007)	●	●	●		5
	Schult et al. (2019)	●		●		
GER-3	Schult et al. (2019)	●		●		2
GEO	Martskvishvili et al. (2020)	●	●	●		3
HRV	Vorkapic (2016)		●	●		2
IND	Akhtar (2018)	●	●	●	●	4
ITA-1	Chiorri et al. (2015)		●	●		2
ITA-2	Chiorri et al. (2015)	●	●	●	●	4
JPN	Oshio et al. (2013)	●	●			7
	Oshio et al. (2014)	●	●			
	Iwasa and Yoshida (2018)	●		●	●	
NOR	Thørrisen et al. (2021)		●	●		2
PER	Azkhosh et al. (2020)	●		●	●	3
POL	Laguna et al. (2014)	●		●	●	3
POR-1	Carvalho et al. (2012)		●			2
POR-2	Brito-Costa et al. (2015)		●			5
	Nunes et al. (2018)	●	●	●	●	
SPA-1	Romero et al. (2012)	●	●	●	●	4
SPA-2	Renau et al. (2013)	●		●	●	3
SPA-3	Renau et al. (2013)	●		●	●	3
TUR	Atak (2013)	●	●	●	●	4

TIPI, Ten-Item Personality Inventory; Con., convergent validity; Str., structural validity; IC, internal consistency; T-R, test–retest reliability.

a See Table 3 for details about versions.

b Convergence with BFI, NEO-FFI and/or NEO-PI-R.

c Score indicating the extent to which each version has been subjected to psychometric inspection.

### 3.3. Convergent validity

Estimates of convergent validity (convergence between identified TIPI versions and NEO-PI-R, NEO-FFI and/or BFI subscales) are presented in Table 5. A more detailed overview of convergent validity estimates is available in Supplementary Table S1.

**Table 5:** Convergent validity (convergence between identified TIPI versions and three other validated FFM instruments).

	Convergence with BFI						Convergence with NEO-FFI						Convergence with NEO-PI-R					
Version^a^	E	A	C	ES	O	All	E	A	C	ES	O	All	E	A	C	ES	O	All
ENG-1	**0.87**	**0.70**	**0.75**	**0.81**	**0.65**	**0.76**	0.48	0.39	**0.66**	**0.61**	**0.52**	**0.53**	**0.65**	**0.59**	**0.68**	**0.66**	**0.56**	**0.63**
BEN	**0.82**	**0.76**	**0.79**	**0.80**	**0.75**	**0.78**	–	–	–	–	–	–	–	–	–	–	–	–
CAT	–	–	–	–	–	–	–	–	–	–	–	–	**0.61**	0.42	**0.63**	**0.55**	0.16	0.47
DUT-1	–	–	–	–	–	–	–	–	–	–	–	–	**0.74**	0.48	**0.66**	**0.70**	0.12	**0.54**
DUT-2	–	–	–	–	–	–	–	–	–	–	–	–	**0.72**	0.49	**0.67**	**0.64**	0.48	**0.60**
DUT-3	**0.68**	**0.59**	**0.66**	**0.70**	**0.68**	**0.66**	–	–	–	–	–	–	–	–	–	–	–	–
FRE	**0.78**	**0.63**	**0.71**	**0.77**	**0.66**	**0.71**	–	–	–	–	–	–	–	–	–	–	–	–
GER-1	–	–	–	–	–	–	0.45	0.08	0.46	**0.66**	**0.23**	0.38	–	–	–	–	–	–
GER-2	–	–	–	–	–	–	**0.57**	**0.64**	**0.67**	**0.77**	0.42	**0.61**	**0.69**	**0.51**	**0.68**	**0.76**	0.41	**0.61**
GER-3	–	–	–	–	–	–	**0.56**	**0.67**	**0.78**	**0.77**	0.48	**0.65**	–	–	–	–	–	–
GEO	**0.85**	**0.50**	**0.75**	**0.85**	**0.51**	**0.69**	–	–	–	–	–	–	–	–	–	–	–	–
IND	**0.80**	**0.61**	**0.66**	**0.68**	0.48	**0.65**	–	–	–	–	–	–	–	–	–	–	–	–
ITA-2	**0.71**	**0.62**	**0.79**	**0.55**	**0.58**	**0.65**	**–**	**–**	**–**	**–**	–	**–**	**–**	–	**–**	**–**	–	**–**
JPN	**0.72**	0.39	0.41	**0.59**	0.46	**0.59**	**0.68**	**0.61**	**0.62**	**0.69**	0.40	**0.60**	**0.65**	0.49	**0.63**	**0.70**	0.46	**0.59**
PER	–	–	–	–	–	–	0.42	0.27	**0.53**	0.10	0.24	0.31	–	–	–	–	–	–
POL	–	–	–	–	–	–	**0.70**	**0.62**	**0.65**	**0.65**	0.38	**0.60**	–	–	–	–	–	–
POR-2	**0.78**	**0.60**	**0.74**	**0.77**	**0.69**	**0.72**	–	–	–	–	–	–	–	–	–	–	–	–
SPA-1	–	–	–	–	–	–	–	–	–	–	–	–	**0.55**	0.36	**0.64**	**0.61**	**0.50**	**0.53**
SPA-2	–	–	–	–	–	–	–	–	–	–	–	–	0.41	0.05	**0.63**	0.40	0.35	0.37
SPA-3	–	–	–	–	–	–	–	–	–	–	–	–	0.45	0.35	**0.70**	0.47	**0.50**	0.49
TUR	**0.58**	0.44	**0.57**	**0.59**	**0.53**	**0.54**	–	–	–	–	–	–	–	–	–	–	–	–
*M*, all	**0.76**	**0.58**	**0.68**	**0.71**	**0.60**	**0.68**	**0.55**	0.47	**0.62**	**0.61**	0.38	**0.53**	**0.61**	0.42	**0.66**	**0.61**	**0.54**	**0.54**

TIPI, Ten-Item Personality Inventory; FFM, Five-factor model of personality (Big Five); BFI, Big Five Inventory (John and Srivastava, 1999); NEO-FFI, NEO Five-Factor Inventory (Costa and McCrae, 1992); NEO-PI-R, NEO Personality Inventory-Revised (Costa and McCrae, 1992); E, extraversion; A, agreeableness; C, conscientiousness; ES, emotional stability; O, openness; *M*, mean; All estimates (correlations) are based on results reported in the included studies. Mean estimates are reported in instances where two or more studies have tested the version’s convergent validity with the same FFM instrument (see Supplementary Table S1 for details); Bold typeface indicates *r* ≥ 0.50.

a See Table 3 for details about versions/translations.

As shown in Table 5, convergence with BFI subscales was tested for 10 versions of the TIPI. Seven of these versions demonstrated acceptable convergence (*r* ≥ 0.50) on all five subscales (ENG-1 in Furnham, 2008 and Gosling et al., 2003; BEN in Islam, 2019; DUT-3 in Denissen et al., 2008; FRE in Storme et al., 2016; GEO in Martskvishvili et al., 2020; ITA-2 in Chiorri et al., 2015; and POR-2 in Nunes et al., 2018). Across all five dimensions, the strongest convergence with BFI subscales was found for BEN (*r* = 0.78 in Islam, 2019), while the weakest was for TUR (*r* = 0.54 in Atak, 2013). The mean convergence for all five dimensions across all 10 versions was *r* = 0.68, well above the *r* ≥ 0.50 threshold.

Convergence with NEO-FFI subscales was tested for 7 versions of the TIPI. None of the TIPI versions reached acceptable convergence on all five dimensions, but for 5 versions the mean subscale correlations reached *r* ≥ 0.50 (ENG-1 in Furnham, 2008; GER-2 and GER-3 in Schult et al., 2019; JPN in Iwasa and Yoshida, 2018; and POL in Laguna et al., 2014). Across all five dimensions, the strongest convergence was found for GER-3 (*r* = 0.65 in Schult et al., 2019), although the correlation for the O subscale did not reach the threshold of *r* ≥ 0.50 (*r* = 0.48). The mean convergence for all five dimensions across all TIPI versions (7 versions) was acceptable (*r* = 0.53).

Convergence with NEO-PI-R subscales was tested for 9 versions of the TIPI. Only one version (ENG-1 in Gosling et al., 2003) demonstrated acceptable convergent validity across all five dimensions. However, for 6 versions, the mean correlations with NEO-PI-R subscales reached *r* ≥ 0.50 (ENG-1 in Gosling et al., 2003; DUT-1 in Hofmans et al., 2008; GER-2 in Muck et al., 2007; JPN in Oshio et al., 2013; and SPA-1 in Romero et al., 2012). The mean convergence for all five dimensions across all TIPI versions (9 versions) was acceptable (*r* = 0.54).

### 3.4. Structural validity

Conclusions from inspections of structural validity of the TIPI versions are presented in Table 6.

**Table 6:** Structural validity of the TIPI versions.

		Satisfactory FFM structure?		
Version^a^	Study	Yes	Partly	No
ENG-1	Ehrhart et al. (2009)	●		
	Metzer et al. (2014)	●		
BEN	Islam (2019), EFA			●
	Islam (2019), CFA	●		
CHI	Shi et al. (2022), EFA			●
	Shi et al. (2022), CFA			●
DUT-1	Hofmans et al. (2008)			●
DUT-2	Hofmans et al. (2008)	●		
FRE	Storme et al. (2016)		●	
GER-1	Herzberg and Brähler (2006)			●
GER-2	Muck et al. (2007)	●		
GEO	Martskvishvili et al. (2020)	●		
HRV	Vorkapic (2016), EFA			●
	Vorkapic (2016), CFA			
IND	Akhtar (2018), EFA			●
	Akhtar (2018), CFA		●	
ITA-1	Chiorri et al. (2015)			●
ITA-2	Chiorri et al. (2015)	●		
JPN	Oshio et al. (2013)	●		
	Oshio et al. (2014)	●		
NOR	Thørrisen et al. (2021)	●		
POR-1	Carvalho et al. (2012)			●
POR-2	Brito-Costa et al. (2015)			●
	Nunes et al. (2018), EFA			●
	Nunes et al. (2018), CFA	●		
SPA-1	Romero et al. (2012)		●	
TUR	Atak (2013), EFA	●		
	Atak (2013), CFA	●		

TIPI, Ten-Item Personality Inventory; FFM, Five-factor model of personality (Big Five); EFA, exploratory factor analysis; CFA, confirmatory factor analysis.

a See Table 3 for details about versions/translations.

Of the 19 versions of TIPI for which structural validity was tested, support for a five-factor structure theoretically corresponding to the FFM dimensions was found for 10 versions (ENG-1 in Gosling et al., 2003 and Metzer et al., 2014; BEN in Islam, 2019; DUT-2 in Hofmans et al., 2008; GER-2 in Muck et al., 2007; GEO in Martskvishvili et al., 2020; ITA-2 in Chiorri et al., 2015; JPN in Oshio et al., 2013, 2014; NOR in Thørrisen et al., 2021; POR-2 in Nunes et al., 2018; and TUR in Atak, 2013). For two of these 10 versions (BEN and POR-2), mixed results were identified. For instance, for the second Portuguese version (POR-2), Nunes et al. (2018) identified an acceptable five-factor structure by means of confirmatory factor analysis, but not through exploratory factor analysis, while Brito-Costa et al. (2015) failed to establish a five-factor structure for POR-2.

For 3 of the 19 versions tested for structural validity (FRE in Storme et al., 2016; IND in Akhtar, 2018; and SPA-1 in Romero et al., 2012), an acceptable five-factor structure was established, but only after making certain statistical adjustments. For instance, Storme et al. (2016) were able to fit a five-factor structure through confirmatory factor analysis of FRE after adjusting the model by including residual covariances.

For six versions of the TIPI (CHI in Shi et al., 2022; DUT-1 in Hofmans et al., 2008; GER-1 in Herzberg and Brähler, 2006; HRV in Vorkapic, 2016; ITA-1 in Chiorri et al., 2015; and POR-1 in Carvalho et al., 2012), attempts of establishing an acceptable five-factor structure failed.

### 3.5. Internal consistency

Estimates of internal consistency for the TIPI versions are presented in Table 7. A more detailed description is shown in Supplementary Table S2.

**Table 7:** Internal consistency of the TIPI versions.

	E			A			C			ES			O			All five domains (*M*)		
Version^a^	α	*r*	S-B	α	*r*	S-B	α	*r*	S-B	α	*r*	S-B	α	*r*	S-B	α	*r*	S-B
ENG-1	0.68	**0.58**	0.51	0.38	0.20	0.38	0.51	0.39	0.53	0.64	0.48	0.64	0.48	0.35	0.34	0.54	0.40	0.48
ENG-2	–	–	0.57	–	–	0.31	–	–	0.60	–	–	0.68	–	–	0.49	–	–	0.53
BEN	0.51	–	–	0.59	–	–	0.63	–	–	0.67	–	–	0.58	–	–	0.60	–	–
CAT	0.67	–	–	0.27	–	–	0.60	–	–	0.67	–	–	0.48	–	–	0.54	–	–
CHI	**0.79**	–	**0.79**	0.12	–	0.13	0.51	–	0.51	0.56	–	0.57	0.32	–	0.32	0.46	–	0.46
FRE	0.69	**0.52**	–	0.22	0.13	–	0.57	0.40	–	0.61	0.44	–	0.39	0.23	–	0.50	0.34	–
GER-1	0.24	–	–	0.33	–	–	0.52	–	–	0.54	–	–	0.41	–	–	0.41	–	–
GER-2	0.60	–	–	0.47	–	–	0.68	–	–	0.68	–	–	0.45	–	–	0.58	–	–
GER-3	**0.71**	–	–	0.54	–	–	0.67	–	–	**0.76**	–	–	0.52	–	–	0.64	–	–
GEO	**0.76**	–	–	0.56	–	–	0.65	–	–	0.69	–	–	0.55	–	–	0.64	–	–
HRV	0.36	–	–	0.13	–	–	0.38	–	–	0.46	–	–	0.41	–	–	0.35	–	–
IND	**0.71**	**0.55**	–	0.31	0.20	–	0.30	0.18	–	0.65	0.49	–	0.34	0.21	–	0.46	0.33	–
ITA-1	0.65	0.48	–	0.23	0.14	–	0.44	0.31	–	0.39	0.24	–	–	–	–	–	–	–
ITA-2	0.69	**0.55**	–	0.38	0.28	–	0.61	0.49	–	0.49	0.36	–	0.48	0.39	–	0.53	0.41	–
JPN	0.56	–	–	0.36	–	–	0.47	–	–	0.52	–	–	0.40	–	–	0.46	–	–
NOR	**0.75**	**0.61**	**0.76**	0.35	0.22	0.36	0.61	0.47	0.64	0.62	0.47	0.64	0.41	0.27	0.41	0.55	0.41	0.56
PER	0.69	–	–	0.40	–	–	0.54	–	–	0.49	–	–	0.45	–	–	0.51	–	–
POL	0.54	–	–	0.41	–	–	0.67	–	–	0.45	–	–	0.42	–	–	0.50	–	–
POR-2	**0.76**	–	–	0.50	–	–	0.38	–	–	0.40	–	–	0.54	–	–	0.52	–	–
SPA-1	0.58	–	–	0.41	–	–	0.53	–	–	0.59	–	–	0.47	–	–	0.52	–	–
SPA-2	0.66	–	–	0.20	–	–	0.56	–	–	0.61	–	–	0.48	–	–	0.50	–	–
SPA-3	0.61	–	–	0.21	–	–	0.53	–	–	0.45	–	–	0.55	–	–	0.47	–	–
TUR	**0.86**	–	–	**0.81**	–	–	**0.84**	–	–	**0.86**	–	–	**0.83**	–	–	**0.84**	–	–
*M*, all	0.64	**0.55**	0.66	0.37	0.19	0.30	0.56	0.37	0.57	0.58	0.41	0.63	0.47	0.29	0.39	0.53	0.38	0.51

TIPI, Ten-Item Personality Inventory; E, extraversion; A, agreeableness; C, conscientiousness; ES, emotional stability; O, openness; *M*, mean; α, Cronbach’s alpha; *r*, inter-item correlation; S-B, Spearman-Brown coefficient. All estimates are based on results reported in the included studies. Mean estimates are reported in instances where two or more studies have tested the version’s internal consistency (see Supplementary Table S2 for details). Bold typeface indicates α ≥0.70, *r* ≥ 0.50 and S-B ≥ 0.70.

a See Table 3 for details about versions/translations.

Of the 23 TIPI versions tested for internal consistency, only the Turkish version (TUR in Atak, 2013) demonstrated acceptable consistency on all five dimensions, with α coefficients ranging from 0.81 to 0.86. The third German version (GER-3 in Schult et al., 2019) showed acceptable internal consistency for two subscales (α_E_ = 0.71 and α_ES_ = 0.76), while eight versions reached an acceptable level on one subscale (E) (ENG-1 in Ehrhart et al., 2009; CHI in Shi et al., 2022; FRE in Storme et al., 2016; GEO in Martskvishvili et al., 2020; IND in Akhtar, 2018; ITA-2 in Chiorri et al., 2015; NOR in Thørrisen et al., 2021; and POR-2 in Nunes et al., 2018). Thirteen of the 23 versions tested for internal consistency failed to demonstrate acceptable consistency on any subscale. Overall, taking all tested versions into account, the α coefficients were highest for E (0.64), followed by ES (0.58), C (0.56), O (0.47) and A (0.37). Across all subscales and tested versions, the average internal consistency for the TIPI was quite low (α = 0.53; *r* = 0.38; S-B = 0.51).

### 3.6. Test–retest reliability

As shown in Table 8, test–retest reliability was tested for 17 versions of the TIPI. Details are presented in Supplementary Table S3.

**Table 8:** Test–retest reliability of the TIPI versions.

Version^a^	E *r*	A *r*	C *r*	ES *r*	O *r*	*M*, all domains *r*
ENG-1	**0.77**	**0.71**	**0.76**	**0.70**	**0.62**	**0.71**
ENG-2	**0.63**	**0.57**	**0.60**	**0.63**	**0.60**	**0.61**
BEN	**0.72**	**0.82**	**0.76**	**0.54**	**0.83**	**0.73**
CAT	**0.85**	**0.69**	**0.81**	**0.82**	**0.70**	**0.77**
DUT-3	**0.75**	**0.58**	**0.71**	**0.73**	**0.70**	**0.69**
FRE^b^	**0.80**	**0.65**	**0.65**	**0.73**	**0.69**	**0.70**
GER-1	**0.83**	**0.67**	**0.83**	**0.84**	**0.65**	**0.76**
IND	**0.85**	**0.79**	**0.71**	**0.74**	**0.75**	**0.77**
ITA-2	**0.87**	**0.81**	**0.90**	**0.79**	**0.89**	**0.85**
JPN^b^	**0.81**	**0.72**	**0.77**	**0.78**	**0.71**	**0.76**
PER	**0.94**^**c**^	**0.91**^**c**^	**0.84**^**c**^	**0.96**^**c**^	**0.94**^**c**^	**0.92**^**c**^
POL	**0.66**	**0.74**	**0.71**	**0.66**	**0.60**	**0.67**
POR-2	**0.90**	**0.71**	**0.82**	**0.78**	**0.83**	**0.81**
SPA-1	**0.79**	**0.52**	**0.69**	**0.83**	**0.78**	**0.72**
SPA-2	**0.81**	**0.61**	**0.77**	**0.76**	**0.72**	**0.73**
SPA-3	**0.55**	**0.64**	**0.78**	**0.56**	**0.59**	**0.62**
TUR	**0.88**	**0.87**	**0.87**	**0.89**	**0.89**	**0.88**
*M*, all	**0.78**	**0.69**	**0.76**	**0.74**	**0.72**	**0.74**

TIPI, Ten-Item Personality Inventory; E, extraversion; A, agreeableness; C, conscientiousness; ES, emotional stability; O, openness; *r*, Pearson correlation coefficient; *M*, mean. Bold typeface indicates *r* ≥ 0.50 and ICC ≥0.60.

a See Table 3 for details about versions.

b Mean estimates based on two reliability tests (see Supplementary Table S3 for details).

c Intraclass correlation coefficient (ICC), not included in mean estimates for all versions (*M*, all).

All 17 versions demonstrated acceptable test–retest reliability on all five dimensions (*r* ≥ 0.50 or ICC ≥0.60). Across all five dimensions, average correlation coefficients ranged from 0.61 for the second English version (ENG-2 in DeBell et al., 2022) to 0.88 for the Turkish version (TUR in Atak, 2013). Across all tested versions, test–retest reliability was highest for the E subscale (*r* = 0.78), and lowest for the A subscale (*r* = 0.69).

### 3.7. Summative evaluation of psychometric properties

A summative overview of psychometric properties associated with the identified TIPI versions is presented in Table 9.

**Table 9:** Summative evaluation of psychometric properties of the TIPI versions.

	Validity		Reliability	
Version^a^	Con.^b^	Str.^c^	IC^d^	T-R^e^
ENG-1	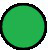	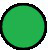	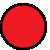	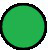
ENG-2	–	–	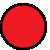	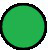
BEN	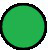	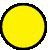	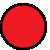	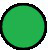
CAT	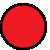	–	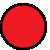	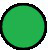
CHI	–	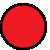	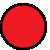	–
DUT-1	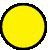	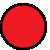	–	–
DUT-2	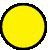	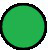	–	–
DUT-3	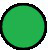	–	–	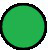
FRE	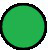	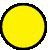	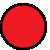	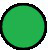
GER-1	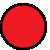	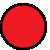	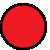	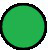
GER-2	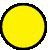	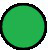	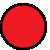	–
GER-3	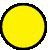	–	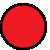	–
GEO	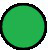	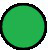	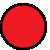	–
HRV	–	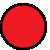	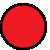	–
IND	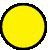	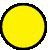	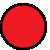	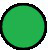
ITA-1	–	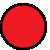	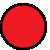	–
ITA-2	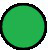	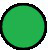	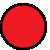	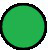
JPN	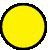	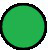	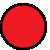	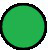
NOR	–	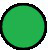	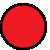	–
PER	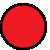	–	–	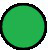
POL	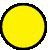	–	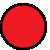	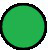
POR-1	–	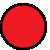	–	–
POR-2	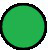	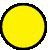	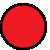	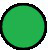
SPA-1	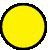	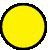	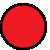	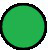
SPA-2	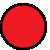	–	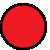	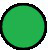
SPA-3	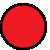	–	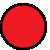	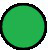
TUR	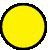	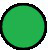	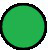	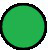

a See Table 3 for details about versions.

b Convergent validity, green indicator, acceptable convergent validity on all five subscales with at least one validated FFM instrument; yellow indicator, mixed results; red indicator, not acceptable convergent validity with any of the FFM instruments, neither separately on the subscales nor on average across subscales.

c Structural validity, green indicator, all tests indicate factor solution in accordance with the FFM; yellow indicator, mixed results; red indicator, no test indicates factor solution in accordance with the FFM.

d Internal consistency, green indicator, at least one acceptable measure of internal consistency on all five subscales separately; red indicator, no test indicates acceptable internal consistency, neither separately for the subscales nor on average across subscales.

e Test-retest reliability, green indicator, acceptable test–retest correlations separately on all five subscales and on average across subscales.

None of the 27 TIPI versions identified in the 29 included studies demonstrated acceptable psychometric properties on all four aspects of validity and reliability. The original English version (ENG-1) and second Italian version (ITA-2) were acceptable with regard to all aspects except internal consistency. The Turkish version (TUR) showed acceptable structural validity, internal consistency and test–retest reliability, but mixed results for convergent validity. The Bengali (BEN), French (FRE) and second Portuguese (POR-2) versions demonstrated acceptable convergent validity and test–retest reliability, but mixed results for structural validity and inappropriate internal consistency. The Japanese version (JPN) was acceptable with regard to structural validity and test–retest reliability, but showed mixed results for convergent validity and inappropriate internal consistency.

## 4. Discussion

The aim of this scoping review was to generate an overview of different versions of the TIPI, and their psychometric properties in terms of two aspects of validity (convergent and structural) and two aspects of reliability (internal consistency and test–retest reliability). A total of 27 TIPI versions were identified in 29 studies, covering 18 different languages. Across versions, and held up against conventional standards of psychometric properties, the TIPI has demonstrated acceptable test–retest reliability, mixed results for convergent and structural validity, and inappropriate internal consistency.

The TIPI is a very brief instrument that intends to capture the breadth of the FFM personality dimensions by means of five subscales, each consisting of two items. It has been emphasized that such an instrument cannot be expected to excel in terms of psychometric properties such as structural validity and internal consistency (Gosling et al., 2003). As noted by Chiorri et al. (2015, p. 110), improved structural validity and internal consistency could quite easily have been achieved by including only “items with a very high correlation (e.g., *r* > 0.70), which, given their unavoidable redundancy, would have undermined content coverage.” Not surprisingly, of the 23 TIPI versions tested for internal consistency, only one version demonstrated acceptable consistency on all five FFM dimensions. It is noteworthy that the vast majority of tests of internal consistency in the included studies utilized Cronbach’s α. Estimates of α are strongly influenced by the number of items in each subscale (Kline, 2000), which is why authors have recommended application of S-B coefficients rather than α coefficients when exploring internal consistency of instruments with brief subscales (e.g., Eisinga et al., 2013). On the other hand, the four TIPI versions actually explored with S-B coefficients did not stand out as substantially more or less internally consistent than those versions investigated by means of alpha (α) and Pearson (*r*) coefficients.

Although acceptable structural validity may not be expected for brief instruments aiming to maximize content coverage, five-factor structures theoretically corresponding with the FFM were identified for 8 of the 19 TIPI versions that were tested for structural validity. As such, some evidence of structural validity of the TIPI does exist, even though results were quite mixed across the different versions.

Given the TIPI’s brevity (five two-item subscales) and purpose (maximization of content coverage across the FFM dimensions), the developers of the instrument emphasized the relevance and importance of convergent validity and test–retest reliability when evaluating psychometric properties of the instrument (Gosling, n.d.; Gosling et al., 2003). All 17 versions that were tested for test–retest reliability displayed acceptable temporal stability on all five FFM dimensions. Hence, this scoping review indicates evidence of acceptable test–retest reliability of the TIPI. However, results for convergent validity were somewhat mixed. Overall, the TIPI displayed strongest convergence with the BFI, with 6 out of 9 tested versions indicating acceptable convergence on all five subscales. Only one version converged satisfactory with the NEO-PI-R on all five dimensions, and none of the tested TIPI versions displayed acceptable convergent validity with all five NEO-FFI dimensions. Still, mean convergences for all tested TIPI versions across all five dimensions reached acceptable levels of correlation (*r* ≥ 0.50) with the BFI, the NEO-FFI, and the NEO-PI-R dimensions.

Given that the TIPI is generally not expected to reach conventional standards of internal consistency and structural validity, some authors have raised the issue of whether the TIPI should be conceptualized as a formative rather than a reflective measure (Myszkowski et al., 2019). The TIPI, in line with other FFM instruments, is generally conceived as a reflective measure, i.e., as a measure consisting of items believed to be correlated and to constitute effects of common latent factors (Markus and Borsboom, 2013). Alternatively, one may conceptualize the TIPI items as samples of particular behaviors rather than correlated effects of common factors, i.e., as a formative measure (Markus and Borsboom, 2013). In an interesting study, Myszkowski et al. (2019) subjected the TIPI to formative measurement evaluation, and concluded that such an approach could be more appropriate for the TIPI than a traditional reflective approach. On the other hand, being developed on the basis of reflective techniques such as exploratory factor analysis (McCrae and John, 1992), one may argue that the FFM is reflective by nature, although the nature of the constructs being measured does not necessarily dictate the approach for instrument development and evaluation (Myszkowski et al., 2019). Further exploration of the TIPI as a potentially formative measurement instrument constitutes a venue for future research.

It should be noted that psychometric shortcomings and variations across different versions of the TIPI may, at least partly, be traced to issues of language and translation procedures. Adequate translation of measurement instruments requires taking psychological, linguistic and cultural considerations into account, preferably within the frame of translation-backtranslation procedures (van de Vijver and Hambleton, 1996; van de Vijver and Tanzer, 2004). The included studies and the TIPI website generally provided scarce information about the translation procedures utilized when the non-English versions of the TIPI were developed. The overall pattern of results from psychometric testing did not differ considerably between English and non-English versions of the TIPI, indicating that item bias due to suboptimal translations may not have played an important role in explaining the psychometric shortcomings identified in this scoping review. However, in some of the included studies, poor item translation necessitated reformulation of items, resulting in two or more versions in the same language. For instance, when exploring the first Dutch version of the TIPI (DUT-1), Hofmans et al. (2008) reported having to rephrase five of the items, resulting in a second Dutch version (DUT-2) that demonstrated more adequate psychometric properties.

In the original validation of the TIPI, the instrument’s developers (Gosling et al., 2003) stated that the TIPI demonstrated adequate psychometric properties and that it stood out as a reasonable proxy for more comprehensive FFM instruments in instances where brief measures are necessary to minimize participants’ response burden. The results of our current scoping review largely corroborates the developers’ initial conclusions. The TIPI stands out as a serviceable measure when researchers want to study personality as one among several constructs, and in instances where it may be appropriate to include individual differences simply as covariates or control variables. Consequently, the TIPI carries the benefit of making personality measurement available for a broad scope of studies, both within and beyond the field of psychology. However, use of the TIPI should be discouraged in studies that primarily aim to explore personality, due to the instrument’s psychometric shortcomings (compared with more comprehensive FFM instruments), and due to the TIPI not being able to measure and distinguish between narrow facet-level constructs that underlie the five broad dimensions in the FFM.

Although this scoping review provides some evidence in favor of the TIPI, it must be noted that psychometric properties and extent of exploration of such properties, varied considerably between the 27 identified versions of the instrument. For some versions, only two psychometric domains were tested. For others, psychometric properties were tested in a single study only. Moreover, more than half of the studies included in this scoping review (15 of 29) were solely based on student samples, while general population samples were included in only 7 out of the 29 studies. Therefore, further psychometric research on the TIPI is warranted.

### 4.1. Methodological considerations

This scoping review is the first secondary research effort focusing on the TIPI. We were able to generate an overview of different versions of the instrument, and their psychometric properties. However, certain limitations should be taken into consideration when interpreting results from our review. Being conducted as a scoping review, we did not subject the included studies to quality assessment. Nor did we analyze results from included studies by means of meta-analyses or other sophisticated techniques usually applied in systematic reviews. Our aim was to scope the literature in order to provide overviews and simple syntheses of results. Such an approach is in line with methodological conventions for scoping reviews, and such reviews do not dictate the application of quality assessments or meta-analyses (Arksey and O'Malley, 2005; Pham et al., 2014; Munn et al., 2018; Peters et al., 2020).

Importantly, we cannot rule out having missed relevant validation studies for some TIPI versions, especially if such studies have been published in non-English languages and journals. It was beyond the scope of our review to identify and evaluate studies published in languages other than English. For instance, the TIPI website (Gosling, n.d.) indicates that the TIPI has been translated into at least 26 different languages, while our scoping review identified studies relating to 18 different languages. It may be that the remaining translations have in fact not been subjected to psychometric testing, or alternatively that test results have been published in non-English articles or in articles that did not specify psychometric testing as a study aim. We encourage researchers to publish their work in English, even though they explore non-English versions of a measurement instrument. By doing this, their work will be readily available for an international audience, enabling secondary research efforts to take international versions into account when exploring psychometric properties of an instrument.

## 5. Conclusion

As the first secondary research effort focusing on the TIPI, this scoping review explored 27 versions of the instrument across 29 studies, covering 18 different languages. Being a brief instrument, the TIPI is indeed characterized by certain psychometric shortcomings. However, this scoping review supports that the TIPI may represent a feasible compromise in instances where it is necessary to strike a balance between maximizing psychometric properties and minimizing survey length. In particular, the TIPI stands out as a serviceable option when researchers want to study personality as one among several constructs, and in instances where it is appropriate to include individual differences simply as covariates or control variables.

## Author contributions

This study was designed by MT and TS. MT analyzed the data and drafted the manuscript. Study selection and data extraction were performed by MT and TS. TS provided scientific input to the different drafts and provided data interpretation. MT and TS made critical revisions and provided intellectual content to the manuscript, approved the final version to be published, and agreed to be accountable for all aspects of this work. All authors contributed to the article and approved the submitted version.

## Conflict of interest

The authors declare that the research was conducted in the absence of any commercial or financial relationships that could be construed as a potential conflict of interest.

## Publisher’s note

All claims expressed in this article are solely those of the authors and do not necessarily represent those of their affiliated organizations, or those of the publisher, the editors and the reviewers. Any product that may be evaluated in this article, or claim that may be made by its manufacturer, is not guaranteed or endorsed by the publisher.

## Supplementary material

The Supplementary material for this article can be found online at: https://www.frontiersin.org/articles/10.3389/fpsyg.2023.1202953/full#supplementary-material

## Footnotes

^1^Emotional stability (ES) is often oppositely termed neuroticism (N).
