# Supplementary material for: The Ten-Item Personality Inventory (TIPI): a scoping review of versions, translations and psychometric properties
Source: Front Psychol. 2023 Jun 26;14:1202953. doi: 10.3389/fpsyg.2023.1202953 (PMC10330951; doi:10.3389/fpsyg.2023.1202953)
Supplement: Supplementary file 1 [file Data_Sheet_1.docx]

Supplementary Material

| **Supplementary Table 1.** Detailed overview of estimates of convergent validity of the TIPI-versions (with three other validated FFM instruments) | | | | | | | |
| --- | --- | --- | --- | --- | --- | --- | --- |
| Version^A^ | Study and instrument | E | A | C | ES | O | *M* All |
| ENG-1 | **BFI:**  Gosling et al. (2003)  **NEO-PI-R:** Gosling et al. (2003)  **NEO-FFI:**  Furnham (2008) | .87  .65  .48 | .70  .59  .39 | .75  .68  .66 | .81  .66  .61 | .65  .56  .52 | .76  .63  .53 |
| BEN | **BFI:**  Islam (2019) | .82 | .76 | .79 | .80 | .75 | .78 |
| CAT | **NEO-PI-R:**  Renau et al. (2013) | .61 | .42 | .63 | .55 | .16 | .47 |
| DUT-1 | **NEO-PI-R:**  Hofmans et al. (2008) | .74 | .48 | .66 | .70 | .12 | .54 |
| DUT-2 | **NEO-PI-R:**  Hofmans et al. (2008) | .72 | .49 | .67 | .64 | .48 | .60 |
| DUT-3 | **BFI:**  Denissen et al. (2008) | .68 | .59 | .66 | .70 | .68 | .66 |
| FRE | **BFI:**  Storme et al. (2016) | .78 | .63 | .71 | .77 | .66 | .71 |
| GER-1 | **NEO-FFI:**  Herzberg and Brähler (2006) | .45 | .08 | .46 | .66 | .23 | .38 |
| GER-2 | **NEO-PI-R:**  Muck et al. (2007)  **NEO-FFI:**  Schult et al. (2019) | .69  .57 | .51  .64 | .68  .67 | .76  .77 | .41  .42 | .61  .61 |
| GER-3 | **NEO-FFI:**  Schult et al. (2019) | .56 | .67 | .78 | .77 | .48 | .65 |
| GEO | **BFI:**  Martskvishvili et al. (2020) | .85 | .50 | .75 | .85 | .51 | .69 |
| IND | **BFI:**  Akhtar (2018) | .80 | .61 | .66 | .68 | .48 | .65 |
| ITA-2 | **BFI:**  Chiorri et al. (2015) | .71 | .62 | .79 | .55 | .58 | .65 |
| JPN | **NEO-PI-R:**  Oshio et al. (2013)  **BFI:**  Oshio et al. (2014)  **NEO-FFI:**  Iwasa and Yoshida (2018), S1  Iwasa and Yoshida (2018), S2  *M (NEO-FFI), JPN* | .65  .72  .70  .66  *.68* | .49  .39  .58  .63  *.61* | .63  .41  .62  .62  *.62* | .70  .59  .68  .70  *.69* | .46  .56  .45  .34  *.40* | .59  .53  .61  .59  *.60* |
| PER | **NEO-FFI:**  Azkhosh et al. (2019) | .42 | .27 | .53 | .10 | .24 | .31 |
| POL | **NEO-FFI:**  Laguna et al. (2014) | .70 | .62 | .65 | .65 | .38 | .60 |
| POR-2 | **BFI:**  Nunes et al. (2018) | .78 | .60 | .74 | .77 | .69 | .72 |
| SPA-1 | **NEO-PI-R:**  Romero et al. (2012) | .55 | .36 | .64 | .61 | .50 | .53 |
| SPA-2 | **NEO-PI-R:**  Renau et al. (2013) | .41 | .05 | .63 | .40 | .35 | .37 |
| SPA-3 | **NEO-PI-R:**  Renau et al. (2013) | .45 | .35 | .70 | .47 | .50 | .49 |
| TUR | **BFI:**  Atak (2013) | .58 | .44 | .57 | .59 | .53 | .54 |
| ^A^See Table 3 in article for details about versions/translations; TIPI = Ten-Item Personality Inventory; FFM = Five-factor model of personality (Big Five); E = extraversion; A = agreeableness; C = conscientiousness; ES = emotional stability; O = openness; *M* = mean; BFI = Big Five Inventory (John and Srivastava, 1999); NEO-FFI = NEO Five-Factor Inventory (Costa and McCrae, 1992); NEO-PI-R = NEO Personality Inventory-Revised (Costa and McCrae, 1992); S1 = study 1; S2 = study 2; All estimates are correlations (Pearson *r*) | | | | | | | |

| **Supplementary Table 2.** Detailed overview of estimates of internal consistency of the TIPI versions | | | | | | | |
| --- | --- | --- | --- | --- | --- | --- | --- |
| Version^A^ | Study and measure | E | A | C | ES | O | *M* All |
| ENG-1 | **Cronbach’s α:**  Gosling et al. (2003)  Ehrhart et al. (2009)  Crede et al. (2012), S1  Crede et al. (2012), S2  Metzer et al. (2014)  *M (α), ENG-1*  **Pearson *r*:**  Ehrhart et al. (2009)  **Spearman-Brown:**  DeBell et al. (2022), S1  DeBell et al. (2022), S2  *M (Spearman-Brown), ENG-1* | .68  .71  .65  .67  .69  *.68*  .58  .47  .54  *.51* | .40  .34  .45  .47  .22  *.38*  .20  .36  .39  *.38* | .50  .56  .55  .45  .50  *.51*  .39  .53  .53  *.53* | .73  .65  .69  .64  .47  *.64*  .48  .59  .68  *.64* | .45  .52  .53  .48  .42  *.48*  .35  .37  .30  *.34* | .55  .56  .57  .54  .46  *.54*  .40  .46  .49  *.48* |
| ENG-2 | **Spearman-Brown:**  DeBell et al. (2022), S1  DeBell et al. (2022), S2  *M (Spearman-Brown), ENG-2* | .63  .50  *.57* | .23  .39  *.31* | .61  .59  *.60* | .66  .69  *.68* | .43  .55  *.49* | .51  .54  *.53* |
| BEN | **Cronbach’s α:**  Islam (2019) | .51 | .59 | .63 | .67 | .58 | .60 |
| CAT | **Cronbach’s α:**  Renau et al. (2013), T1  Renau et al. (2013), T2  *M (α), CAT* | .62  .71  *.67* | .28  .26  *.27* | .59  .60  *.60* | .67  .66  *.67* | .53  .43  *.48* | .54  .53  *.54* |
| CHI | **Cronbach’s α:**  Shi et al. (2022)  **Spearman-Brown:**  Shi et al. (2022) | .79  .79 | .12  .13 | .51  .51 | .56  .57 | .32  .32 | .46  .46 |
| FRE | **Cronbach’s α:**  Storme et al. (2016)  **Pearson r:**  Storme et al. (2016) | .69  .52 | .22  .13 | .57  .40 | .61  .44 | .39  .23 | .50  .34 |
| GER-1 | **Cronbach’s α:**  Herzberg and Brähler (2006) | .24 | .33 | .52 | .54 | .41 | .41 |
| GER-2 | **Cronbach’s α:**  Muck et al. (2007)  Schult et al. (2019)  *M (α), GER-2* | .57  .63  *.60* | .42  .52  *.47* | .66  .69  *.68* | .67  .68  *.68* | .54  .35  *.45* | .57  .57  *.58* |
| GER-3 | **Cronbach’s α:**  Schult et al. (2019) | .71 | .54 | .67 | .76 | .52 | .64 |
| GEO | **Cronbach’s α:**  Martskvishvili et al. (2020) | .76 | .56 | .65 | .69 | .55 | .64 |
| HRV | **Cronbach’s α:**  Vorkapic (2016) | .36 | .13 | .38 | .46 | .41 | .35 |
| IND | **Cronbach’s α:**  Akhtar (2018)  **Pearson *r*:**  Akhtar (2018) | .71  .55 | .31  .20 | .30  .18 | .65  .49 | .34  .21 | .46  .33 |
| ITA-1 | **Cronbach’s α:**  Chiorri et al. (2015), S1  **Pearson *r*:**  Chiorri et al. (2015), S1 | .65  .48 | .23  .14 | .44  .31 | .39  .24 | -  - | -  - |
| ITA-2 | **Cronbach’s α:**  Chiorri et al. (2015), S2  Chiorri et al. (2015), S3  Chiorri et al. (2015), S4  *M* (*α*), *ITA-2*  **Pearson *r*:**  Chiorri et al. (2015), S2  Chiorri et al. (2015), S3  *M (r), ITA-2* | .72  .70  .64  *.69*  .56  .54  *.55* | .38  .44  .31  *.38*  .27  .29  *.28* | .67  .57  .60  *.61*  .55  .43  *.49* | .50  .56  .40  *.49*  .33  .39  *.36* | .58  .50  .35  *.48*  .43  .35  *.39* | .57  .55  .46  *.53*  .43  .40  *.41* |
| JPN | **Cronbach’s α:**  Iwasa and Yoshida (2018), S1  Iwasa and Yoshida (2018), S2  *M (α), JPN* | .57  .54  *.56* | .29  .42  *.36* | .49  .44  *.47* | .51  .52  *.52* | .47  .33  *.40* | .47  .45  *.46* |
| NOR | **Cronbach’s α:**  Thørrisen et al. (2021)  **Pearson *r*:**  Thørrisen et al. (2021)  **Spearman-Brown:**  Thørrisen et al. (2021) | .75  .61  .76 | .35  .22  .36 | .61  .47  .64 | .62  .47  .64 | .41  .27  .41 | .55  .41  .56 |
| PER | **Cronbach’s α:**  Azkhosh et al. (2019) | .69 | .40 | .54 | .49 | .45 | .51 |
| POL | **Cronbach’s α:**  Laguna et al. (2014) | .54 | .41 | .67 | .45 | .42 | .50 |
| POR-2 | **Cronbach’s α:**  Nunes et al. (2018), T1  Nunes et al. (2018), T2  *M (α), POR-2* | .72  .79  *.76* | .39  .61  *.50* | .45  .31  *.38* | .43  .37  *.40* | .60  .48  *.54* | .52  .51  *.52* |
| SPA-1 | **Cronbach’s α:**  Romero et al. (2012), T1  Romero et al. (2012), T2  *M (α), SPA-1* | .54  .61  *.58* | .38  .44  *.41* | .54  .51  *.53* | .59  .59  *.59* | .48  .46  *.47* | .51  .52  *.52* |
| SPA-2 | **Cronbach’s α:**  Renau et al. (2013), T1  Renau et al. (2013), T2  *M (α), SPA-2* | .71  .61  *.66* | .08  .32  *.20* | .48  .63  *.56* | .51  .70  *.61* | .51  .44  *.48* | .46  .54  *.50* |
| SPA-3 | **Cronbach’s α:**  Renau et al. (2013) | .61 | .21 | .53 | .45 | .55 | .47 |
| TUR | **Cronbach’s α:**  Atak (2013) | .86 | .81 | .84 | .86 | .83 | .84 |
| ^A^See Table 3 in article for details about versions/translations; TIPI = Ten-Item Personality Inventory; E = extraversion; A = agreeableness; C = conscientiousness; ES = emotional stability; O = openness; *M* = mean; S1 = study 1; S2 = study 2; T1 = time 1; T2 = time 2 | | | | | | | |

| **Supplementary Table 3.** Detailed overview of estimates of test-retest reliability of the TIPI versions | | | | | | | |
| --- | --- | --- | --- | --- | --- | --- | --- |
| Version^A^ | Study | E | A | C | ES | O | *M* All |
| ENG-1 | Gosling et al. (2003) | .77 | .71 | .76 | .70 | .62 | .71 |
| ENG-2 | DeBell et al. (2022) | .63 | .57 | .60 | .63 | .60 | .61 |
| BEN | Islam (2019) | .72 | .82 | .76 | .54 | .83 | .73 |
| CAT | Renau et al. (2013) | .85 | .69 | .81 | .82 | .70 | .77 |
| DUT-3 | Denissen et al. (2008) | .75 | .58 | .71 | .73 | .70 | .69 |
| FRE | Storme et al. (2016), 3W  Storme et al. (2016), 6W  *M, FRE* | .78  .82  *.80* | .62  .68  *.65* | .58  .72  *.65* | .70  .76  *.73* | .69  .68  *.69* | .67  .73  *.70* |
| GER-1 | Herzberg and Brähler (2006) | .83 | .67 | .83 | .84 | .65 | .76 |
| IND | Akhtar (2018) | .85 | .79 | .71 | .74 | .75 | .77 |
| ITA-2 | Chiorri et al. (2015) | .87 | .81 | .90 | .79 | .89 | .85 |
| JPN | Iwasa and Yoshida (2018), S1  Iwasa and Yoshida (2018), S2  *M, JPN* | .84  .78  *.81* | .74  .70  *.72* | .77  .76  *.77* | .78  .78  *.78* | .75  .67  *.71* | .78  .74  *.76* |
| PER | Azkhosh et al. (2019) | .94* | .91* | .84* | .96* | .94* | .92* |
| POL | Laguna et al. (2014) | .66 | .74 | .71 | .66 | .60 | .67 |
| POR-2 | Nunes et al. (2018) | .90 | .71 | .82 | .78 | .83 | .81 |
| SPA-1 | Romero et al. (2012) | .79 | .52 | .69 | .83 | .78 | .72 |
| SPA-2 | Renau et al. (2013) | .81 | .61 | .77 | .76 | .72 | .73 |
| SPA-3 | Renau et al. (2013) | .55 | .64 | .78 | .56 | .59 | .62 |
| TUR | Atak (2013) | .88 | .87 | .87 | .89 | .89 | .88 |
| ^A^See Table 3 in article for details about versions/translations; TIPI = Ten-Item Personality Inventory; E = extraversion; A = agreeableness; C = conscientiousness; ES = emotional stability; O = openness; *M* = mean; 3W = 3-week time interval; 6W = 6-week time interval; S1 = study 1; S2 = study 2; *Intraclass correlation coefficient (ICC); All other estimates are correlations (Pearson *r*) | | | | | | | |
